# Global genome nucleotide excision repair is organized into domains that promote efficient DNA repair in chromatin

**DOI:** 10.1101/gr.209106.116

**Published:** 2016-10

**Authors:** Shirong Yu, Katie Evans, Patrick van Eijk, Mark Bennett, Richard M. Webster, Matthew Leadbitter, Yumin Teng, Raymond Waters, Stephen P. Jackson, Simon H. Reed

**Affiliations:** 1 Division of Cancer and Genetics, School of Medicine, Cardiff University, Cardiff, CF14 4XN, United Kingdom;; 2 Wellcome Trust/Cancer Research UK Gurdon Institute, University of Cambridge, Cambridge, CB2 1QN, United Kingdom

## Abstract

The rates at which lesions are removed by DNA repair can vary widely throughout the genome, with important implications for genomic stability. To study this, we measured the distribution of nucleotide excision repair (NER) rates for UV-induced lesions throughout the budding yeast genome. By plotting these repair rates in relation to genes and their associated flanking sequences, we reveal that, in normal cells, genomic repair rates display a distinctive pattern, suggesting that DNA repair is highly organized within the genome. Furthermore, by comparing genome-wide DNA repair rates in wild-type cells and cells defective in the global genome–NER (GG-NER) subpathway, we establish how this alters the distribution of NER rates throughout the genome. We also examined the genomic locations of GG-NER factor binding to chromatin before and after UV irradiation, revealing that GG-NER is organized and initiated from specific genomic locations. At these sites, chromatin occupancy of the histone acetyl-transferase Gcn5 is controlled by the GG-NER complex, which regulates histone H3 acetylation and chromatin structure, thereby promoting efficient DNA repair of UV-induced lesions. Chromatin remodeling during the GG-NER process is therefore organized into these genomic domains. Importantly, loss of Gcn5 significantly alters the genomic distribution of NER rates; this has implications for the effects of chromatin modifiers on the distribution of mutations that arise throughout the genome.

DNA, the key molecule of heredity, is susceptible to damage to its structure because it is continually exposed to the deleterious effects of normal cellular metabolic processes and external genotoxic stresses, such as ultraviolet (UV) radiation and chemical damage (Friedberg 2003). Thousands of lesions occur every day in the DNA of each of our cells, the immediate implications of which include disruption of DNA replication and cell division as well as defective gene regulation. Long-term effects include the introduction of DNA mutations, which alter the genetic information of the cell. Repair of damaged DNA is therefore fundamental to the maintenance of genome stability (Holmquist and Gao 1997). Whole-exome sequencing studies of various human cancer types (The Cancer Genome Atlas Research Network et al. 2013) identified tumor-specific somatic mutations and multiple mutational signatures associated with different cancer types (Alexandrov et al. 2013a). The causes of these mutational signatures fall into two groups: environmental mutagens, such as UV light or polycyclic aromatic hydrocarbons from cigarette smoke, or defects in DNA repair pathways (Nik-Zainal et al. 2012; Alexandrov et al. 2013a,b). Collectively, these observations demonstrate the importance of understanding how genetic damage is formed and efficiently repaired in cells.

Nucleotide excision repair (NER) acts on a spectrum of DNA damage that have the common property of distorting the DNA double helix. Over 30 polypeptides are involved in the basic NER reaction. Two damage-recognition pathways exist: the transcription coupled repair pathway (TC-NER) that operates on the transcribed strands of transcribing genes and involves RNA polymerase II in damage recognition; and the global genome repair pathway (GG-NER) that operates on all DNA, including nontranscribed and repressed regions of the genome, involving a unique subset of proteins in the early stages of DNA damage recognition (Fousteri and Mullenders 2008). Following the initial stages of DNA damage detection, these two pathways converge and utilize the same DNA repair proteins. The majority of yeast NER genes have well-conserved structural and/or functional human homologs, and the main features of both the GG-NER and TC-NER pathways are evolutionarily conserved (Hoeijmakers 1993, 1994).

In the nucleus, DNA is packaged into the nucleoprotein complex of chromatin. At present, how NER operates on naked DNA is well understood, but our knowledge of how it operates in chromatin is still emerging (Adam et al. 2015). Determining how DNA damage is sensed and removed from DNA packaged into chromatin is central to our understanding of genome stability and its effects on human health. Recent advances are providing important insights into such responses (Adam et al. 2015; Polo 2015). We identified the *Saccharomyces cerevisiae* protein complex of Rad7, Rad16, and Abf1, required for GG-NER in yeast, referred to as the GG-NER complex. We showed that efficient GG-NER requires Abf1 to be bound to specific DNA binding sites (Reed et al. 1999), which can be found at hundreds of locations throughout the yeast genome (Yu et al. 2009). The Rad16 protein is a member of the SWI/SNF super-family of chromatin remodeling factors. Proteins in this super-family contain conserved ATPase motifs and are subunits of protein complexes with chromatin-remodeling activity (Flaus and Owen-Hughes 2011). Since Rad16 operates on repressed and nontranscribed regions of the genome during GG-NER, it has long been assumed that its role might involve chromatin remodeling (Verhage et al. 1994), conceivably, to improve access to damaged DNA. Rad16 also contains a C3HC4-type RING domain, which is important in ubiquitin E3 ligase proteins. We have previously reported that the GG-NER complex also has E3 ubiquitin ligase activity involving the Cul3 and Elc1 proteins (Pintard et al. 2004; Willems et al. 2004; Gillette et al. 2006).

Previously, we investigated how the yeast GG-NER complex remodels chromatin by examining events at a single genetic locus (Yu et al. 2011). This work established that the complex promotes UV-induced chromatin remodeling necessary for DNA repair by recruiting the histone acetyl-transferase (HAT) Gcn5 onto chromatin, which promotes increased histone H3 acetylation levels that, in turn, alter chromatin structure (Yu et al. 2011). These observations demonstrated that the GG-NER complex promotes the UV-induced chromatin remodeling necessary for DNA repair at the genetic locus examined.

In the present study, we carry out an expanded investigation of these parameters to examine how the GG-NER process is organized throughout the yeast genome. To tackle this issue, we developed a genome-wide DNA repair assay based on ChIP-chip, referred to as 3D-DIP-Chip (Teng et al. 2011; Powell et al. 2015). The method permits the calculation of the relative repair rates at individual sites throughout the genome. This is a novel way of examining DNA repair rates in wild-type and various mutant strains and of measuring the distribution of genomic DNA repair rates. We also measured the chromatin binding of the individual GG-NER factors, HAT occupancy, and histone H3 acetylation levels in chromatin, before and after UV irradiation, to understand how these events are organized in the genome. Our observations may explain how mutations in novel cancer genes involved in regulating chromatin structure may alter patterns of genomic stability during tumorigenesis.

## Results

### The GG-NER complex promotes efficient repair of UV-induced DNA damage in nontranscribed genomic regions

**Figure 1.**
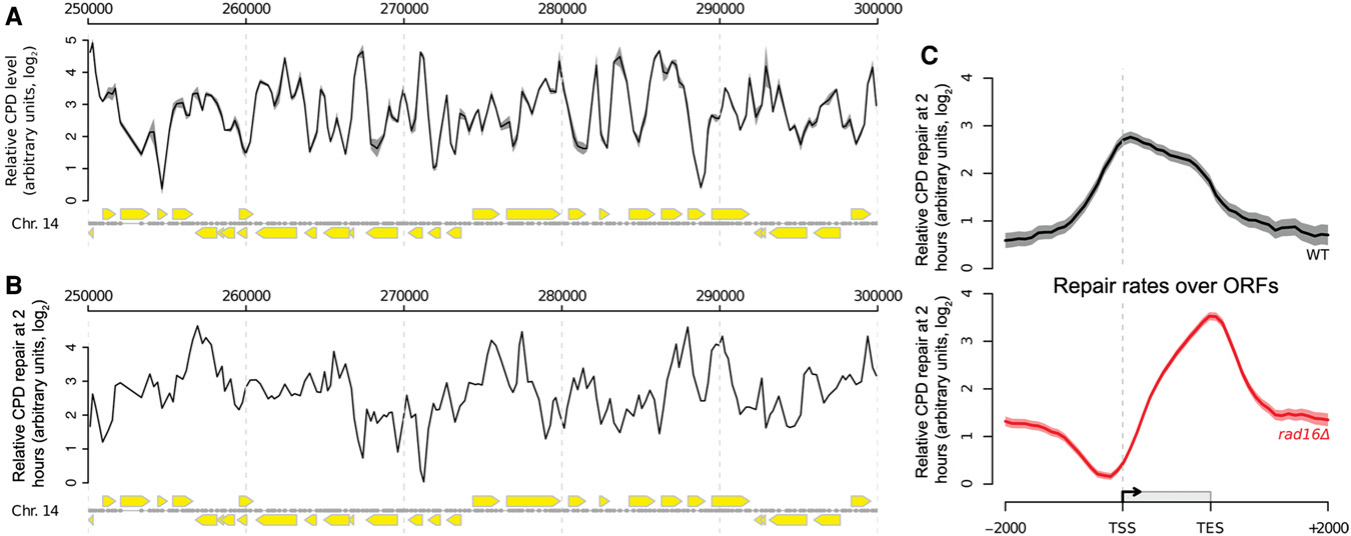
Genome-wide UV-induced DNA repair is organized around gene structure. (*A*) A linear genome plot of a section of Chromosome 14 showing 3D-DIP-Chip results from wild-type cells. The black line shows the mean (*n* = 3) CPD level observed immediately after UV irradiation (100 J/m^2^, shading highlights the SEM). Gray dots indicate the positions of microarray probes. Yellow arrows indicate ORF positions and their direction of transcription. CPD levels are plotted as arbitrary units on the *y*-axis. (*B*) CPD repair rates displayed in a linear genome plot. The black line shows the mean of CPD levels 120 min post-UV (*n* = 2) subtracted from the mean at 0 min post-UV shown in *A*. Annotations are as described in *A*. (*C*) Relative rates of CPD repair around ORF structures. Solid lines show the mean of CPD repair rates in wild-type (*n* = 3, black line) and *rad16*Δ cells (*n* = 2, red line). Shaded areas indicate the SD, with CPD levels plotted as arbitrary units on the *y*-axis.

We used 3D-DIP-Chip (Teng et al. 2011; Powell et al. 2015) to measure UV-induced DNA damage throughout the yeast genome at different time points after UV irradiation, to investigate the role of GG-NER in promoting removal of this damage. We previously developed the R software package Sandcastle (Bennett et al. 2015) for the analysis of this data, and it has been used to create the plots shown here. As previously (Teng et al. 2011), we observed a heterogeneous distribution of CPDs throughout the genome immediately after UV irradiation (Fig. 1A). To calculate relative rates of cyclobutane pyrimidine dimer (CPD) removal at different locations throughout the genome, we repeated the 3D-DIP-Chip procedure with DNA from cells that had been allowed 2 h of repair and then subtracted these values from CPD levels immediately after UV irradiation to generate a genome-wide pattern of relative DNA repair rates. It is important to note that this assay measures DNA damage and repair on both strands of the DNA molecule, meaning that the relative repair rates observed reflect the combined activity of the GG-NER and TC-NER pathways. We selected the 2-h time point to measure the relative DNA repair rates, as this represents a time of active repair. As shown previously (Teng et al. 2011; Powell et al. 2015), we observed a heterogeneous distribution of relative DNA repair rates for the removal of CPDs in relation to their linear arrangement in the genome (Fig. 1B). Therefore, to examine the distribution of repair rates in relation to gene structure, we produced composite gene plots of open reading frames (ORFs) and their flanking regions (described in Supplemental Fig. S1A). ORFs ranging from 500 to 1500 bp were used to generate composite plots of relative DNA repair rates, including DNA sequences up to 2 kbp upstream and downstream. This represents ∼85% of the yeast genome. It is important to note that the profile-plotting function in Sandcastle ensures that no region of the genome is represented more than once in these plots. This feature is important in preventing the duplication of genomic data where the regions plotted overlap (as illustrated in Supplemental Fig. S1A). We refer to this style of figure as a “composite plot.”

Presenting wild-type relative repair rate data as composite plots reveals a uniform distribution of repair rates in intergenic regions, with a gradual increase in repair rates in the promoter regions of genes, reaching a peak at transcription start sites (TSSs) and the 5′ end of ORFs (Fig. 1C, black line). Enhanced rates of repair are observed throughout the ORFs, with rates gradually reducing toward transcription end sites (TES), with further reduction in intergenic rates downstream from the TES. It has previously been established that the enhanced rate of repair in ORFs is due to the combined activity of GG-NER and TC-NER operating on actively transcribing strands (Hu et al. 2015). To examine this, we analyzed CPD repair rates in the 15% of the lowest expressed or silent genes in the genome, as defined by global gene expression data for wild-type cells (Zhou et al. 2015). Supplemental Figure S1B shows that these genes have little or no enhanced rates of repair in ORFs. This is in contrast to the remaining 85% of genes in the genome that are transcribed at higher rates (Supplemental Fig. S1C).

To determine the contribution of the GG-NER pathway to the distribution of relative CPD repair rates in wild-type cells, we examined events in *RAD16* deleted cells. In the absence of Rad16, there is a marked alteration in the distribution of relative repair rates around ORFs (Fig. 1C, red line). The greatest reduction in relative repair rates as compared to rates in cells expressing Rad16 is observed in the intergenic promoter regions, with rates becoming less affected within ORFs before reducing again in the intergenic regions downstream from TESs (Fig. 1C, red line). This altered pattern is due to the absence of the GG-NER pathway, resulting in the loss of repair in nontranscribing DNA. It is important to note that the relative rates of repair we show do not represent absolute levels of lesion removal, as often described in other DNA repair assays. Instead, they represent the distribution of the various repair rates measured throughout the genome. For this reason, we plotted relative repair rate data from different strains separately, shown as arbitrary units on a log_2_-scale of the *y*-axis to indicate this, as shown in Figure 1C. These results demonstrate that the GG-NER complex generates the pattern of DNA repair rates observed in wild-type cells and suggests a structure to the repair process. We next considered how GG-NER is organized in the genome of wild-type cells.

### GG-NER is organized and initiated from Abf1 binding sites found at thousands of locations in the yeast genome

**Figure 2.**
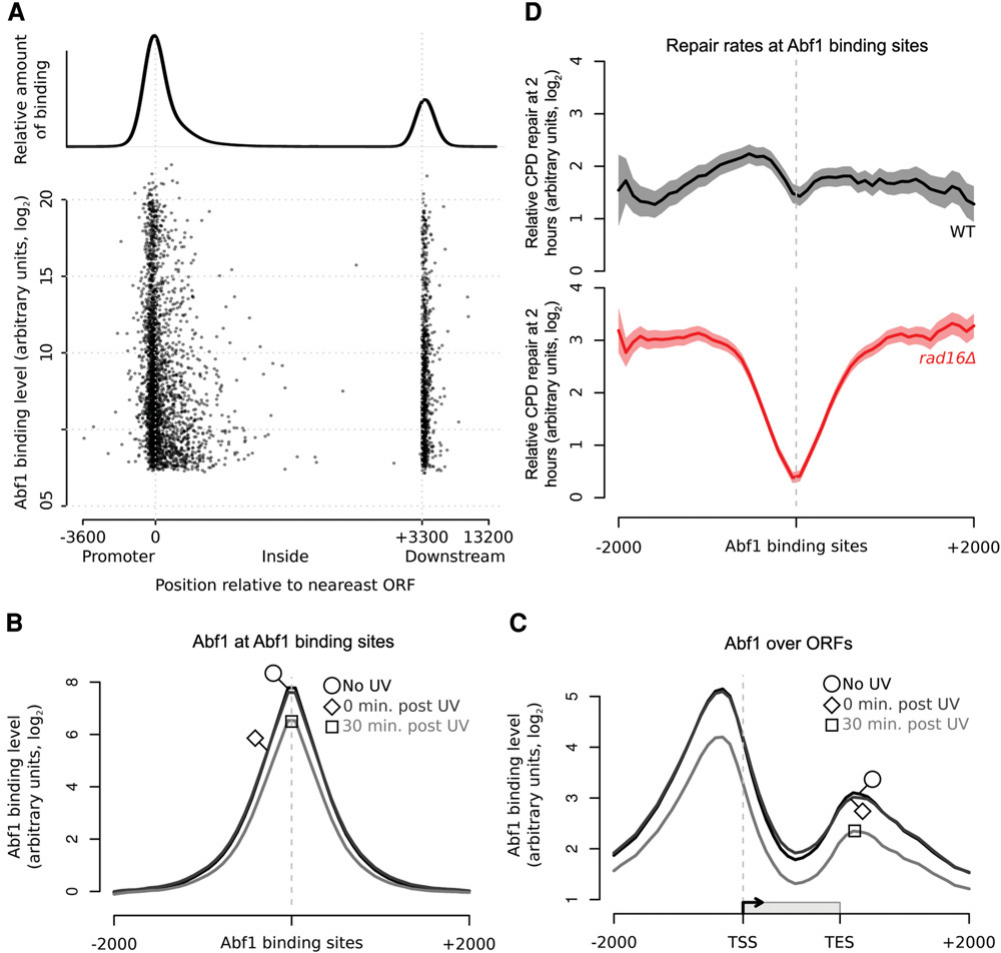
GG-NER is organized from Abf1 binding sites, and Abf1 occupancy does not change significantly in response to UV. (*A*) The positions of Abf1 binding relative to ORFs. Abf1 binding levels at the ∼3800 detected binding sites are shown. Each binding site is represented by a single data point, with the overall relative amount of binding throughout the region shown *above*. (*B*) ChIP-chip data for Abf1 binding. Data are shown for unirradiated (black, circle), 0 min post-UV (dark gray, diamond), and 30 min post-UV (light gray, square) cells. Solid lines show the means of three data sets per time point. (*C*) As in *B*, plotted around ORF structure. (*D*) Relative CPD repair rates around Abf1 binding sites. The data depicted in Figure 1C are used here to plot the relative rates of CPD removal around Abf1 binding sites in wild-type (black) and *rad16*Δ cells (red). Solid lines show mean CPD repair rates in wild-type (*n* = 3, black line) and *rad16*Δ cells (*n* = 2, red line). The shaded areas show the SEM and SD, with CPD levels plotted as arbitrary units on the *y*-axis.

Abf1 has a wide range of functions in processes including transcription (Buchman et al. 1988; Miyake et al. 2004; Yarragudi et al. 2007; Schlecht et al. 2008), gene silencing (Boscheron et al. 1996; Zou et al. 2006; Zhang et al. 2012), replication (Rhode et al. 1992), and NER (Yu et al. 2004, 2009). We have reported that binding of the Abf1 component of the GG-NER complex to one of its DNA recognition sequences promotes efficient GG-NER both in vitro and in vivo (Yu et al. 2009). Using standard chromatin immunoprecipitation (ChIP) and qPCR, we demonstrated Abf1 binding at a single Abf1 consensus binding site called the “I silencer,” located at the yeast *HMLALPHA* locus (Yu et al. 2009). Mutation of this DNA consensus site caused loss of Abf1 and GG-NER complex binding and reduced GG-NER efficiency extending from the mutated Abf1 DNA binding site. These data suggested that, prior to UV damage, the GG-NER complex might be localized at specific Abf1 binding sites. To determine whether GG-NER is organized from these sites, we used ChIP-chip to measure chromatin occupancy of each component of the GG-NER complex before and at times during the 2-h repair period after UV damage. We first measured genome-wide Abf1 binding, which found around 3800 sites distributed throughout the yeast genome. An example of a linear genomic plot of Abf1 binding in a section of Chromosome 14 is shown in Supplemental Figure S2. Other workers have investigated Abf1 binding using different methods (Yarragudi et al. 2007; Ganapathi et al. 2011; Kasinathan et al. 2014). Our study found similar binding profiles to the most recently reported study, which employed a next-generation sequencing-based (NGS) method (Zentner et al. 2015). We demonstrate that these sites are located predominantly in intergenic regions, mainly in promoters and, to a lesser extent, TESs (Fig. 2A). Plotting Abf1 occupancy at its binding sites shows no marked change in response to UV, exhibiting only a slight reduction in overall binding 30 min after UV irradiation (Fig. 2B). To assess the genomic distribution of Abf1 in more detail, we plotted its binding in relation to gene structure. This shows that Abf1 is highly enriched in promoter proximal regions (Fig. 2C, black line). Within ORFs, Abf1 occupancy is much lower, while elevated levels of occupancy are detected downstream from the TES. These results show that Abf1 occupancy and its overall distribution in relation to ORF structure do not change markedly after UV irradiation (Fig. 2C, dark gray line). A small loss in overall Abf1 occupancy, evenly distributed across the ORF, is detected at 30 min after UV irradiation (Fig. 2C, light gray line). We conclude that Abf1 is stably bound at intergenic regions of the genome and does not change in response to UV irradiation.

To determine whether GG-NER is organized from these Abf1 binding sites, we plotted our DNA repair rate data for both wild-type and GG-NER-defective *RAD16*-deleted cells as composite plots centered on the Abf1 binding sites (Fig. 2D). This revealed that the relative rates of repair in *RAD16-*deleted cells are markedly reduced around Abf1 binding sites compared to wild-type cells. Importantly, plotting the distribution of DNA repair rates at an equal number of randomly generated simulated ORFs reveals an even distribution of repair rates in both wild-type and *RAD16*-deleted cells (Supplemental Fig. S3). These observations confirm that Abf1 binding sites play a significant role in organizing GG-NER in the genome.

### The GG-NER complex protein Rad7 localizes to Abf1 binding sites

**Figure 3.**
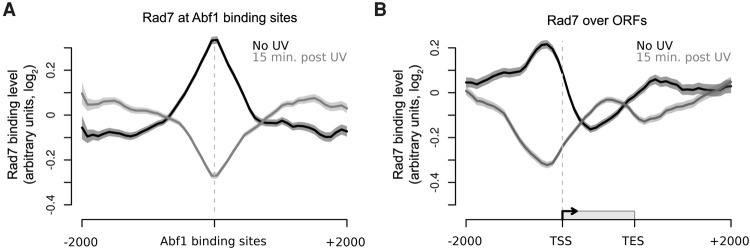
The colocalization of the GG-NER factor Rad7 in chromatin at Abf1 binding sites and its redistribution in response to UV irradiation. (*A*) Rad7 binding data around detected Abf1 binding sites in the absence of UV (black) and 15 min post-UV (gray). Solid lines show the means of three data sets, and shaded areas show the SEM. (*B*) As in *A*, plotted around ORF structure.

Our previous studies, examining events at the *HMLALPHA* locus (Yu et al. 2009), indicated that the GG-NER complex occupies the chromatin at this Abf1 binding site, where it promotes efficient DNA repair. We considered whether GG-NER complex binding at multiple Abf1 binding sites organizes and primes the genome for efficient repair. To investigate this, we used ChIP-chip to measure the genome-wide occupancy of Rad7 and plotted the data at Abf1 binding sites. This reveals a strong enrichment of Rad7 occupancy at these sites (Fig. 3A, black line), extending our previous observations (Yu et al. 2009) and demonstrating that Rad7 colocalizes at multiple Abf1 binding sites in the absence of UV damage. Next, we investigated the effect of UV irradiation on Rad7 binding. In wild-type cells, Rad7 occupancy at Abf1 binding sites is markedly reduced 15 min after UV, but complete loss of occupancy from chromatin does not occur (Fig. 3A; cf. black and gray lines). Displaying the data as composite gene plots orientates the Abf1 binding sites in relation to ORFs (Fig. 3B). The UV-induced redistribution of Rad7 can then be discerned, revealing that Rad7 dissociates from Abf1 binding sites in promoter and downstream regions and redistributes predominantly into ORFs and upstream promoter regions.

### The Rad7 and Rad16 proteins colocalize with Abf1 in the genome

**Figure 4.**
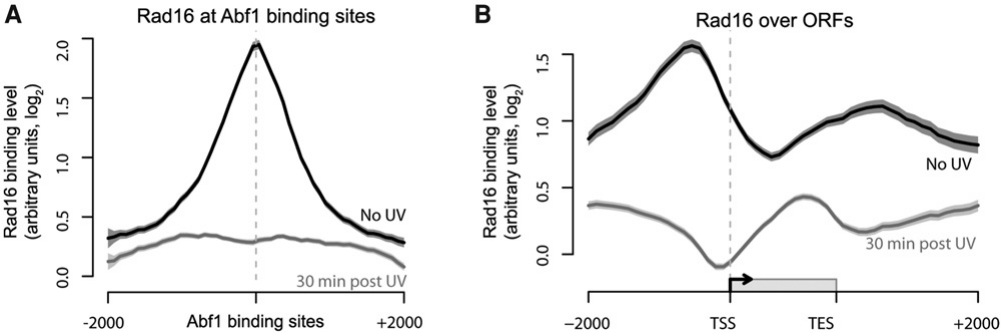
Rad16 associates with chromatin surrounding Abf1 binding sites and is redistributed in response to UV similar to Rad7. (*A*) Rad16 binding data around Abf1 binding sites for unirradiated (black) and 30 min post-UV (gray) cells. Solid lines show the means of three data sets, and shaded areas show the SEM. (*B*) As in *A*, plotted around ORF structure.

To establish whether the genomic occupancy of Rad16 is similar to that of Rad7 and Abf1, we performed ChIP-chip for Rad16 and plotted the resulting data around Abf1 binding sites (Fig. 4A, black and gray lines). This confirmed the colocalization of these proteins at these sites. We noted UV-induced loss of Rad16 occupancy from Abf1 binding sites 30 min after damage, akin to that observed for Rad7 at 15 min (Fig. 3A). Similar observations are made when examining events as composite gene plots (Fig. 4B). Slightly reduced levels of Rad16 chromatin occupancy are observed 30-min after UV irradiation. As anticipated, Rad16 distribution around ORFs prior to UV irradiation is very similar to that of Abf1 (Fig. 2C, black line) and Rad7 protein binding (Fig. 3B, black line). Notably, the Rad16 redistribution observed 30 min after UV damage during DNA repair is very similar to that of Rad7 at 15 min. Finally, we established that the distribution of Rad7 is dependent on the GG-NER complex by performing ChIP-chip for Rad7 in a *RAD16-*deleted strain. As shown in Supplemental Figure S4A, displaying the data as composite gene plots reveals that the normal pattern of Rad7 distribution prior to UV irradiation depends on Rad16. (Supplemental Fig. S4A; cf. light and dark red lines). These observations were confirmed when we examined events in the context of GG-NER complex occupancy at Abf1 binding sites (Supplemental Fig. S4B). Collectively, these results demonstrate that, prior to UV irradiation, the Rad7 and Rad16 components of the GG-NER complex locate at Abf1 binding sites found in intergenic regions of the genome, particularly in promoter and downstream regions of genes. In response to UV damage, a complex of Rad7 and Rad16 redistributes away from Abf1 binding sites to occupy locations within the ORFs during the DNA repair period.

### Rad16 genomic occupancy depends on its ATPase and RING domain functions

**Figure 5.**
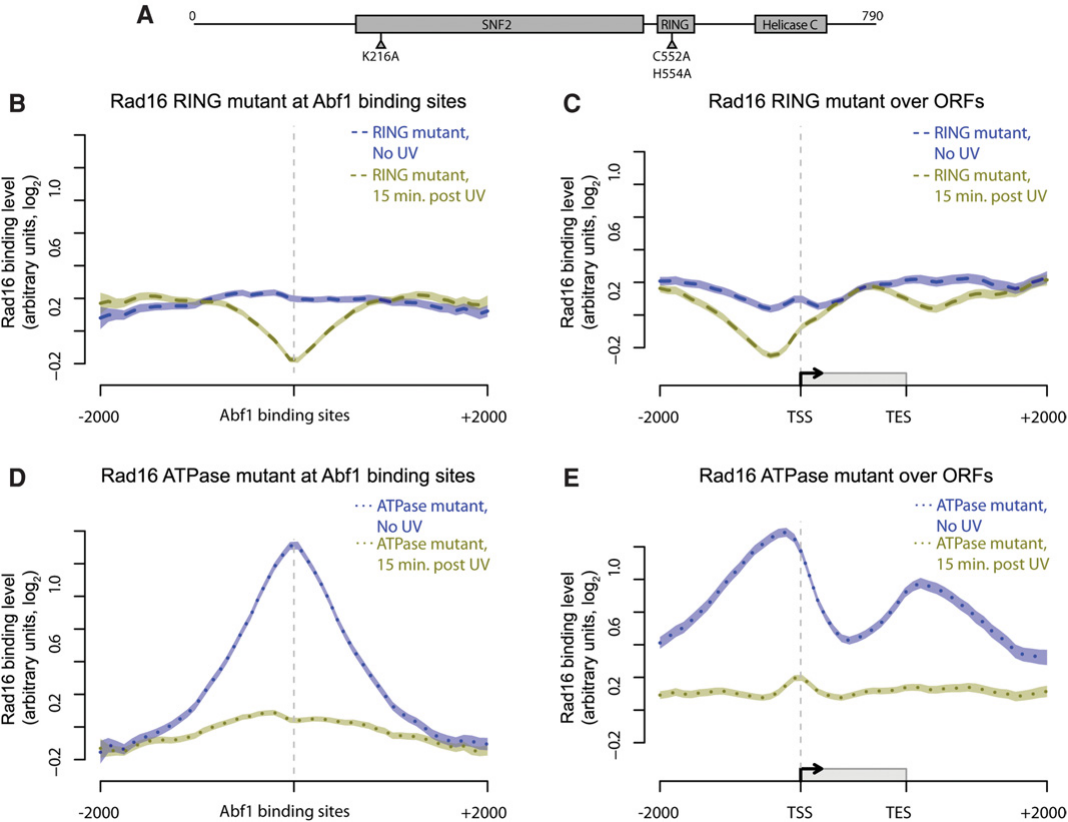
The activity of both the ATPase and RING domain of Rad16 determine its chromatin occupancy before and after UV irradiation. (*A*) Representation of the linear structure of Rad16. The amino acids targeted by the point mutations introduced in the ATPase (K216A) and RING domains (C552A, H554A) are highlighted. (*B*–*E*) Composite plots of Rad16 chromatin occupancy in the mutants described. Mutated Rad16 binding data around Abf1 binding sites and ORF structures in the absence of UV irradiation (dark blue) and 15 min after UV irradiation (green) are shown here. The Rad16 RING mutant binding data (dashed lines) are shown in *B* (around Abf1 binding sites) and *C* (around ORFs). The binding data for Rad16 ATPase domain mutant (dotted lines) are shown in *D* (around Abf1 binding sites) and *E* (around ORFs). Lines show the means of three data sets per condition, and shaded areas show the SEM.

We next investigated which Rad16 functions are responsible for the genomic distribution of the GG-NER complex before and after UV radiation. Rad16 contains within its structure two functional regions that contribute to efficient GG-NER: two SWI/SNF ATPase domains; and an E3 ubiquitin ligase RING domain (Fig. 5A). It has previously been reported that individually inactivating these domains reduces repair rates and results in intermediate UV sensitivity, while mutating both domains generates UV sensitivity equivalent to a *rad16* null strain (Ramsey et al. 2004; Yu et al. 2011). We measured the genomic occupancy of Rad16 in strains containing point mutations in the ATPase domain, the RING domain, or both domains together. Strains expressing these mutated genes produce full-length Rad16 proteins (Supplemental Fig. S5A) that can associate with chromatin, as shown by Western blot analysis (Supplemental Fig. S5B). The distribution of Rad16 at Abf1 binding sites before and after UV-irradiation in wild-type cells (Fig. 4A) is lost in the ATPase/RING double-mutant strain (Supplemental Fig. S6A, dark blue and green solid lines, respectively). Similar results are seen in the composite gene plot (Supplemental Fig. S6B), confirming the loss of the expected pattern of Rad16 distribution observed in wild-type cells. These data establish that the distribution of Rad16 before and its redistribution after UV irradiation during the repair period depends on functional ATPase and RING domains.

Inactivating the E3 ligase results in the loss of the wild-type Rad16 occupancy at Abf1 binding sites in the absence of UV irradiation, revealing an even chromatin distribution (Fig. 5B,C, dark blue dashed line). However, in response to UV, some redistribution of this mutant Rad16 protein still occurs (Fig. 5B,C; cf. dark blue lines with green lines), indicating that the intact ATPase domains promote UV-induced redistribution of Rad16. In contrast, an ATPase-mutated Rad16 protein shows a normal genomic distribution in the absence of UV damage (cf. Fig. 4A,B with Fig. 5D,E, dark blue dotted lines), while a large reduction in Rad16 chromatin occupancy is observed in response to UV irradiation. However, the UV-induced redistribution of Rad16 into the ORFs observed in wild-type cells does not occur (Fig. 5D,E, green lines). We conclude that Rad16 E3 ubiquitin ligase activity is required for establishing and maintaining Rad16 occupancy at Abf1 binding sites prior to UV irradiation, while the ATPase activity is dispensable for this. ATPase activity is required for Rad16 redistribution in response to UV damage.

### The GG-NER complex regulates genome-wide distribution of Gcn5 chromatin occupancy before and after UV irradiation

**Figure 6.**
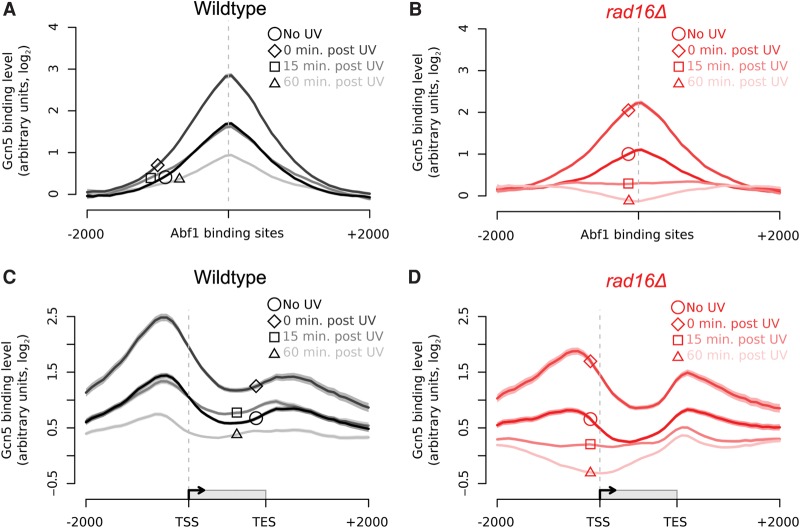
Gcn5 is recruited to Abf1 binding sites and ORFs in response to UV in a Rad16-dependent manner. (*A*) Gcn5 binding data in wild-type cells around Abf1 binding sites for unirradiated (black; circle highlight), 0 min post-UV (dark gray; diamond highlight), 15 min post-UV (mid-gray; square highlight), and 60 min post-UV (light gray; triangle highlight) cells. Solid lines show means (*n* = 3, 3, 2, and 3, respectively), and shaded areas show the SEM (SD for *n* = 2). (*B*) Gcn5 binding data in *rad16*Δ cells around Abf1 binding sites for unirradiated (red; circle highlight), 0 min post-UV (dark pink; diamond highlight), 15 min post-UV (mid-pink; square highlight), and 60 min post-UV (light pink; triangle highlight) cells. Solid lines show the means of two data sets per time point, and shaded areas show the SD. (*C*) As in *A*, plotted around ORF structure (see Fig. 1C). (*D*) As in *B*, plotted around ORF structure (see Fig. 1C).

UV-induced chromatin modifications contribute to efficient repair at the *MFA2* locus during GG-NER, through Gcn5-dependent hyperacetylation of histone H3K9 and H3K14 (Yu et al. 2005). In our previous work, we noted that UV-induced acetylation occurs independently of the core NER factors Rad4 and Rad14, demonstrating that functional NER is not required for this activity. However, we found that the *RAD7* and *RAD16* genes are required for UV-induced acetylation of histone H3K9/K14 at the *MFA2* locus and that this was achieved by the GG-NER complex controlling chromatin occupancy of the HAT Gcn5 (Yu et al. 2005). We also reported that this process promotes chromatin remodeling, making the chromatin more accessible to restriction enzyme digestion (Yu et al. 2011). To determine how the GG-NER complex controls Gcn5 chromatin occupancy, we performed ChIP-chip experiments for Gcn5 binding. This established that Gcn5 is enriched at Abf1 binding sites prior to UV irradiation (Fig. 6A, black line, circle highlight), similar to the components of the GG-NER complex (Figs. 2C, 3A, 4A). Gcn5 occupancy in the vicinity of these sites increases immediately following UV irradiation (Fig. 6A, dark gray line, diamond highlight) and gradually reduces after 15 min (Fig. 6A, mid-gray line, square highlight), with further reduction in Gcn5 occupancy observed 60 min after UV irradiation (Fig. 6A, light-gray line, triangle highlight). Importantly, although occupancy is reduced, enrichment of Gcn5 around Abf1 binding sites is retained during this period (Fig. 6A). To investigate whether the GG-NER complex plays a role in regulating the UV-induced change in Gcn5 occupancy, we measured Gcn5 binding in the absence of Rad16. The results show that, prior to UV irradiation, Gcn5 binding is similar to that seen in wild-type cells, but at slightly lower levels (Fig. 6B, solid red line, circle highlight). We observed an initial UV-induced recruitment of Gcn5 to Abf1 binding sites in the absence of Rad16, but at lower levels of enhancement than that observed in wild-type cells. This indicates a limited contribution of the GG-NER complex to wild-type levels of Gcn5 recruitment (Fig. 6B, dark pink line, diamond highlight). However, 15 min after UV irradiation, Gcn5 is no longer enriched at these sites in the *RAD16*-deleted strain compared to wild-type cells (Fig. 6B, mid-pink line, square highlight), and occupancy is further reduced after 60 min (Fig. 6B, light pink line, triangle highlight). Figure 6C reveals a similar Gcn5 distribution in relation to ORFs to that of Abf1 and the GG-NER factors prior to UV irradiation. Figure 6D shows that in *RAD16*-deleted cells, Gcn5 occupancy is reduced, predominantly in the vicinity of the promoter proximal Abf1 binding sites compared to wild-type cells at the 15- and 60-min time points during DNA repair. We also plotted the combined data for Gcn5 binding in wild-type and *RAD16*-deleted cells at each of the different time points measured during DNA repair (Supplemental Fig. S7A–D). We conclude from these results that the GG-NER complex regulates Gcn5 occupancy in chromatin in promoter proximal domains prior to and following UV irradiation.

### The GG-NER complex regulates the UV-induced genomic distribution of histone H3 acetylation

**Figure 7.**
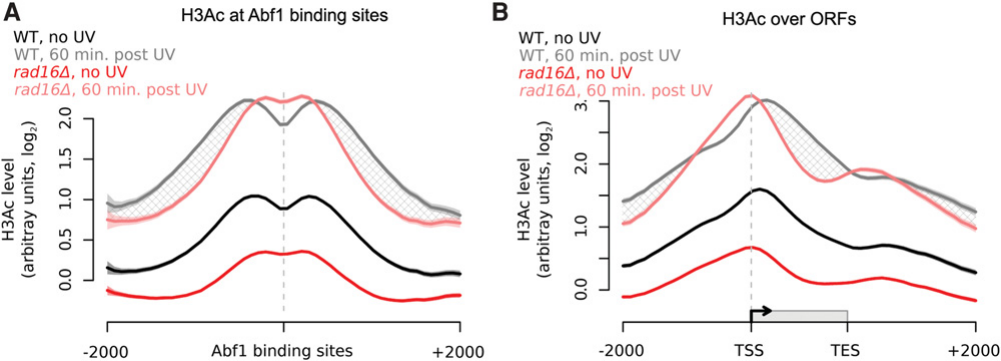
Histone H3 acetylation levels in response to UV irradiation in wild-type and *rad16*Δ cells depend on the GG-NER complex. (*A*) Histone H3 acetylation in wild-type (*n* = 5, black/gray) and *rad16*Δ (*n* = 3, red/pink) cells in response to UV irradiation around Abf1 binding sites. The hatched areas define the genomic regions of GG-NER-dependent UV-induced histone H3 acetylation. Solid lines show the mean, and shaded areas show the SEM. (*B*) As in *A*, plotted around ORF structure.

Having established the genomic distribution of Gcn5 occupancy in chromatin before and after UV irradiation, we next investigated how the histone modification catalyzed by this HAT is distributed within the genome. In wild-type cells, we observe a distinctive “m-shaped” pattern for this epigenetic mark around Abf1 binding sites (Fig. 7A, black line). Histone H3 acetylation (H3Ac) reaches a maximum ∼300 bp either side of Abf1 binding sites and reduces at positions located further away. The lower levels of histone H3Ac centered at Abf1 binding sites is likely caused by the absence of histones at these predominantly nucleosome-free regions (NFRs) (Hartley and Madhani 2009; Ozonov and van Nimwegen 2013). In the absence of UV irradiation, H3Ac is distributed around genes in a similar fashion to the occupancy of Gcn5 in wild-type cells (cf. Fig. 7B, black line, and Fig. 6C, black line). In response to UV irradiation, an increase in histone H3 acetylation is detected, with a maximum enrichment observed at ∼500 bp on either side of the Abf1 binding sites (Fig. 7A, gray line), and the characteristic “m-shaped” pattern of histone modification is retained. However, in a *RAD16*-deleted strain, lower levels of histone H3 acetylation are observed in the absence of UV irradiation compared to wild-type cells (Fig. 7A, dark red line), in line with the reduced Gcn5 occupancy we observed previously (Fig. 6B,D). This indicates that Rad16 plays a role in determining the basal level and distribution of histone H3 acetylation in the absence of DNA damage. In response to UV irradiation, induction of histone H3Ac can still be observed (Fig. 7A, light red line), corresponding to the Rad16-independent recruitment of Gcn5 to Abf1 binding sites described in the previous section (Fig. 6A,B). This may be related to other Gcn5-dependent processes such as transcription. We note that the Rad16-dependent UV-induced increase in histone H3 acetylation observed in wild-type cells corresponds to the redistribution of the GG-NER complex components Rad7 (Fig. 3B) and Rad16 (Fig. 4B). Importantly, the UV-induced distribution of histone H3Ac around Abf1 binding sites and ORFs (Fig. 7B, gray and light red lines, respectively, shaded areas) is significantly different in *RAD16*-deleted cells compared to wild-type cells. The shaded area in Figure 7B identifies the GG-NER complex-dependent histone H3 acetylation in response to UV irradiation. We conclude that the GG-NER complex directs the UV-induced propagation of histone H3 acetylation by regulating the residency of Gcn5 in the genomic domains described.

### Defective UV-induced chromatin remodeling results in altered patterns of DNA repair rates throughout the genome

**Figure 8.**
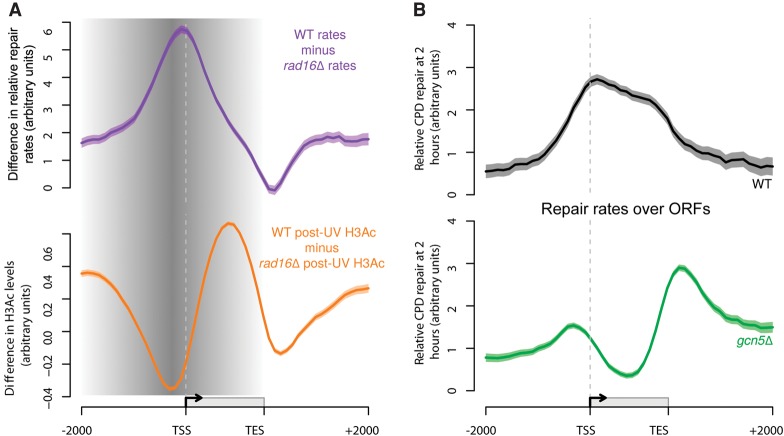
The GG-NER pathway coordinates lesion removal by controlling UV-induced histone H3 acetylation in genomic domains around Abf1 binding sites. (*A*) Rad16-dependent repair (purple line) and UV-induced H3Ac (orange line) are shown here. The shading highlights the domain where these processes are controlled by the GG-NER complex, initiated from sites of Abf1 binding. (*B*) Relative rates of CPD removal around ORF structures in wild-type (*n* = 3, black) and *gcn5*Δ (*n* = 2, green) cells. Solid lines show the mean of relative CPD repair rates levels, with the shaded areas highlighting the SEM or SD, respectively. CPD levels are plotted as arbitrary units on the *y*-axis.

We previously reported that UV-induced histone H3 acetylation promotes chromatin remodeling that is necessary for efficient GG-NER (Yu et al. 2005). In the present study, we have shown how the GG-NER complex regulates this process and how these events are organized within the yeast genome. In Figure 1C, we demonstrated the effect on the distribution of relative genomic DNA repair rates when the GG-NER pathway is abrogated in *rad16*-mutated cells. In Figure 8A (upper panel, purple line), we have plotted the difference in the distribution of relative DNA repair rates between the wild-type and *rad16*-mutant strains, to define the genomic regions affected by loss of the GG-NER pathway. This reveals that relative DNA repair rates are most affected in the promoter regions, upstream of TSSs, where Abf1 is predominantly located. This effect on repair extends in both directions into the upstream promoter, as well as into the ORFs. A similar analysis plotting the difference between UV-induced histone H3 acetylation in wild-type and *RAD16*-deleted cells (Fig. 8A, lower panel, orange line) reveals the reciprocity between relative DNA repair rates and UV-induced histone H3 acetylation levels in the absence of GG-NER. This defines the genomic domains from which GG-NER organizes and initiates chromatin remodeling and repair, highlighted by gray shading. Strikingly, the genomic regions that exhibit defective histone H3 acetylation in GG-NER-defective cells align with the regions of altered relative DNA repair rates observed in these cells (Fig. 8A). To investigate the importance of histone H3 acetylation on the distribution of relative genomic DNA repair rates, we deleted the gene for the HAT *GCN5.* This results in the complete loss of UV-induced histone H3Ac at K9/K14 observed in wild-type cells, confirming the central role of this histone modifier in promoting UV-induced H3Ac in the genome (Supplemental Fig. S8). Finally, we measured the distribution of relative DNA repair rates of UV-induced DNA damage in the absence of Gcn5. Figure 8B compares the relative repair rates in *GCN5*-deleted cells (lower panel, green line) to those in wild-type cells (upper panel, black line) in the context of gene structure. These data establish that the genomic distribution of DNA repair rates is disrupted in the absence of the HAT Gcn5. This confirms the importance of the GG-NER complex in regulating the UV-induced, Gcn5-catalyzed histone H3 acetylation on the wild-type distribution of relative genomic DNA repair rates.

## Discussion

This report provides new insights into understanding the processes that govern genome stability and how these events are organized within the genome. We reveal that the genome is organized in such a way that ensures the efficient removal of DNA damage by the GG-NER pathway. We show that Abf1 binding sites provide locations from which GG-NER is organized to promote efficient genomic DNA repair. To demonstrate this, we mapped relative genomic DNA repair rates in relation to the genomic occupancy of the GG-NER complex components, both before and during a 2-h DNA repair period, following exposure of cells to UV irradiation. We focused on repair of UV-induced cyclobutane pyrimidine dimers by the GG-NER pathway in yeast. Using 3D-DIP-Chip (Teng et al. 2011; Powell et al. 2015), we generated genome-wide DNA damage and relative repair rate profiles in wild-type and mutant yeast strains. This showed that, in wild-type cells, both the initial pattern of CPD induction and the subsequent distribution of their relative DNA repair rates are heterogeneously distributed throughout the genome when viewed as a linear representation of the chromosomes. Importantly, however, presenting such data as composite gene plots around ORFs revealed a level of organization of genomic repair rates in wild-type cells that was previously unknown. We noted enhanced rates of CPD removal within the ORFs in wild-type cells, which is consistent with the known contribution of the TC-NER pathway to the rapid removal of lesions from the transcribed strand of active genes (Mellon et al. 1987). A similar observation was made in a recent study measuring genomic DNA repair using the NGS-based method XR-seq in human cells (Hu et al. 2015; Adar et al. 2016). To examine the effect of removing the GG-NER pathway, we measured DNA repair rates in *RAD16*-deleted cells and observed a significantly altered distribution of relative genomic DNA repair rates (Fig. 1C, lower panel). The altered genomic DNA repair rate profile observed represents the contribution of the TC-NER pathway, which remains intact in these mutant cells. It's important to note that the representation of the data from these DNA damage and repair experiments describes only the distribution of the relative rates of repair throughout the genome and not the absolute levels of lesion removal, as is typically reported for other DNA repair assays.

We have considered how the pattern of genomic DNA repair rates observed might be established in the genome. First, we examined Abf1 binding in the absence of UV damage and observed ∼3800 peaks distributed throughout the genome. The majority of these sites are located in the promoter region of genes, close to the TSSs, and a second, less abundant group can be found at the 3′ end of ORFs near the TES. This demonstrates that the vast majority of Abf1 binding sites are located in intergenic, nontranscribed regions of the genome. To determine whether these sites represent locations from which GG-NER is organized, we plotted genomic DNA repair rates for wild-type cells against GG-NER defective *RAD16*-deleted cells in relation to all Abf1 binding sites. This revealed significantly reduced repair in the vicinity of Abf1 binding sites in these GG-NER-defective cells, suggesting that GG-NER is organized from these sites. Genomic Abf1 distribution does not change markedly in response to UV irradiation. Similar experiments for the Rad7 and Rad16 components of the GG-NER complex show that they colocalize with Abf1 at multiple Abf1 binding sites in the absence of UV irradiation. This demonstrates that the GG-NER complex is chromatin-bound in the absence of DNA damage. However, during repair, and in contrast to Abf1 itself, a striking loss of Rad7 and Rad16 occupancy is seen at Abf1 binding sites, followed by a distinctive redistribution of these proteins extending into the ORFs. These observations demonstrate that the Abf1 component of the GG-NER complex anchors the repair factors Rad7 and Rad16 to its binding sites in the absence of DNA damage. This establishes the presence of GG-NER nucleation sites at these genomic positions, priming the genome for efficient repair. Future studies will focus on the mechanism of the UV-induced dissolution of the GG-NER complex and its role in chromatin remodeling during repair.

By studying the effects of inactivating mutations in key domains of Rad16, we found that the RING E3 ligase motif was important for the pre-UV irradiation distribution of Rad16 observed in wild-type cells, whereas the ATPase domain is dispensable for this. This suggests that ubiquitylation of an as yet undefined target protein is necessary for normal positioning of the complex in the genome in the absence of DNA damage. Potential targets for ubiquitylation include histones, which may tether the GG-NER complex to the chromatin at Abf1 binding sites. In this regard, we note that the UV-DDB complex, which is involved in GG-NER in human cells, is a component of an E3 ubiquitin ligase that ubiquitylates histone H2A in response to UV damage (Kapetanaki et al. 2006; Lan et al. 2012). In contrast, we found that the ATPase domain is required for the post-UV redistribution of Rad16 into the ORFs seen in wild-type cells. This observation is consistent with the presence of ATPase motifs in Rad16 that are required for the DNA translocase activity of the complex (Yu et al. 2004).

**Figure 9.**
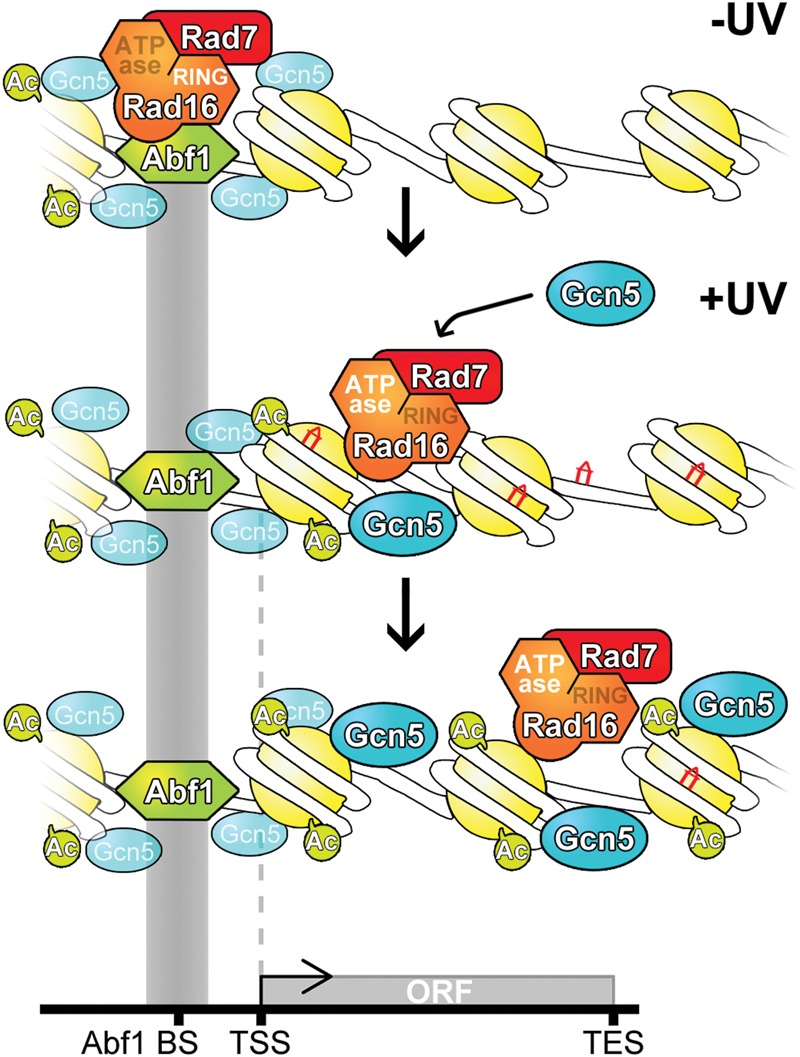
Model to illustrate how GG-NER is organized in the yeast genome. (*Top* panel) In undamaged cells, the GG-NER complex is located at multiple Abf1 binding sites predominantly in the promoter regions of genes. This occupancy is dependent on the RING domain of the Rad16 protein. The enrichment of GG-NER-independent basal levels of Gcn5 can be detected at these sites. (*Middle* panel) In response to UV irradiation, the GG-NER complex dissociates from the Abf1 component at Abf1 binding sites. This process depends on the activity of the ATPase domain in Rad16. Concomitantly, the HAT Gcn5 is recruited onto the chromatin with its increased levels and distribution dependent on the Rad7-Rad16 GG-NER complex. (*Bottom* panel) During this process, histone H3 acetylation is increased over a domain defined by the redistribution of the Rad7-Rad16 proteins from Abf1 binding sites. This mechanism drives the chromatin remodeling necessary for the efficient repair of UV damage.

Our previous studies suggested that the GG-NER complex controls UV-induced histone H3 acetylation by regulating recruitment of Gcn5 onto the chromatin (Yu et al. 2011). Examining Gcn5 occupancy on a genomic scale in our current study revealed how the GG-NER complex controls its occupancy on the chromatin at the correct genomic locations necessary to promote efficient GG-NER. We found that retention of Gcn5 at the genomic locations observed depends on UV-induced redistribution of the GG-NER complex during a 1-h repair period after UV damage. Consistent with a potential UV-induced interaction between the GG-NER complex and Gcn5, we also found that the GG-NER complex controls UV-induced histone H3 acetylation at the same genomic locations. Deletion of *RAD16* results in lower levels of histone H3 acetylation in the absence of UV damage, highlighting a role for the GG-NER complex in setting basal levels of histone H3 acetylation in the genome. Whether this affects cellular processes outside of NER remains unknown. Finally, we established that the genomic regions most affected by loss of GG-NER correspond to the regions most affected by UV-induced, GG-NER-dependent histone H3 acetylation. We conclude that the GG-NER complex regulates the chromatin structure in the vicinity of Abf1 binding sites in response to UV irradiation by controlling the occupancy of the HAT Gcn5 on the chromatin and the UV-induced histone H3 acetylation status at these sites in the genome, as described in the model shown in Figure 9.

We have shown that deleting histone modifiers such as the HAT *GCN5* significantly alters the distribution of repair rates seen in wild-type cells. This observation is striking because *GCN5*-deleted cells are moderately UV-sensitive and only partially defective in overall repair of UV lesions. However, our experiments reveal that the genomic distribution of relative DNA repair rates in these cells is markedly altered. We speculate that this could alter the distribution of UV-induced genomic mutations. If so, this may have important implications for genomic stability during tumorigenesis, because cancer cells frequently display altered regulation of chromatin structure.

Recent reports have begun to measure and decipher the nonrandom nature of the mutational patterns that shape the somatic cancer genome of different cancers types. These include efforts to explain the causes of these mutation patterns based on our current knowledge of DNA damage and repair mechanisms (Haradhvala et al. 2016). Most recently, genomic DNA repair rates have been correlated with the incidence of mutations in skin and other cancers, suggesting that cancer-associated mutations occur in regions of the genome that are more difficult to repair. Recent evidence also suggests that, in human cells, binding of transcription factors at DNase I-hypersensitive sites in gene promoters results in lower levels of DNA repair and higher rates of mutation. This suggests that NER may also be organized in the human genome (Adar et al. 2016; Perera et al. 2016; Sabarinathan et al. 2016). Collectively, these studies demonstrate the importance of understanding the genomic organization of DNA repair mechanisms in chromatin.

## Methods

### Strains and plasmids

The yeast strains and plasmids used in this study have been described previously (Yu et al. 2011) and are listed in Table 1. Mutations were confirmed by sequencing, and successful epitope tagging was confirmed by Western blotting.

**Table 1.**
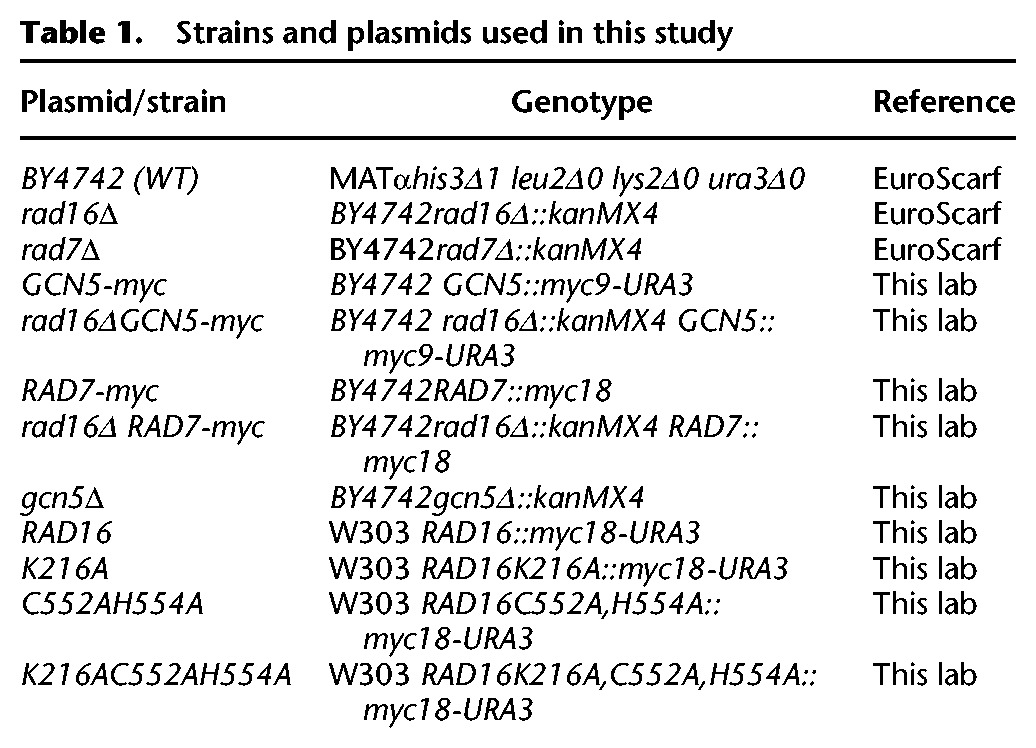
Strains and plasmids used in this study

### UV irradiation, yeast cell culture, and crosslinking

Yeast cells were grown and UV-irradiated as described previously (Yu et al. 2011). After the indicated repair time in YPD, cells were crosslinked with formaldehyde. Cells were harvested and resuspended in cold PBS. For Rad7 affinity capture using ChIP, a double crosslinking method is required using DMA (dimethyl adipmidatedihydrochloride). For details, see Supplemental Methods.

### Chromatin preparation

Chromatin extracts were prepared as described previously (Teng et al. 2011; Yu et al. 2011). Briefly, cells were washed and collected by centrifugation and prepared for lysis by bead beating. The whole-cell extract was then sonicated with a Bioruptor (Diagenode) as described previously (Yu et al. 2011), after which the chromatin extract was collected by centrifugation.

### Chromatin immunoprecipitation

ChIP was performed as described previously (Yu et al. 2011; Powell et al. 2015). Prewashed pan-mouse or anti-rabbit IgG Dynabeads were incubated with the respective antibody. Dynabeads were collected, washed, and resuspended in PBS-BSA (0.1%), after which sonicated chromatin was added to each sample. Following incubation, samples were washed and eluted from the Dynabeads. Crosslinking was reversed and the DNA purified using the PureLink Quick PCR Purification kit (Invitrogen). For details, see Supplemental Methods.

### DNA preparation and IP for CPD detection

DNA was prepared and sonicated as described previously (Teng et al. 2011). IP was conducted as described in the previous section (“Chromatin immunoprecipitation”) with the exception of using an antibody for CPD IP (2 µg per sample of anti-thymine dimer clone KTM53 [Kamiya Biomedical Company]). Following IP, all samples were processed in the same way for microarray.

### Removal of CPDs prior to microarray preparation, and real-time PCR

CPDs were removed from the UV-treated samples prior to PCR amplification and microarray hybridization. The PreCR DNA Repair kit (New England Biolabs) removed much DNA damage, including CPDs.

### DNA preparation and microarray hybridization

Samples were prepared for microarray hybridization as detailed in the Agilent Technologies Yeast ChIP-on-chip protocol, version 9.2. The IP and input samples were combined and applied to Agilent yeast whole-genome microarrays. Microarrays were scanned, and the image was processed using Agilent Feature Extraction software. Analysis of the data was conducted using Sandcastle (Bennett et al. 2015) in R, version 3.2.4 (R Core Team 2016).

### Data normalization

Data from each experiment were normalized using the “normalize” function in Sandcastle (Bennett et al. 2015). The full Sandcastle normalization procedure was applied to the individual protein binding and H3Ac data sets. Only the quantile normalization step was applied to each set of replicates of the CPD data sets, because these data are not suitable for the full Sandcastle normalization procedure.

### Data analysis

The composite plots shown in this paper were created using the “profilePlot” function of Sandcastle. Plots around Abf1 binding sites were created using peaks detected in the untreated Abf1 binding data sets using the “enrichmentDetection” function. Plots over ORFs were created using data downloaded from the Ensembl databases using the “loadAnnotation” function. Full details of these procedures are described in Bennett et al. (2015).

## Data access

ChIP-chip data from this study have been submitted to the EBI ArrayExpress (http://www.ebi.ac.uk/arrayexpress/) under accession number E-MTAB-4641.

## Supplementary Material

Supplemental Material
