# Supplementary material for: Global genome nucleotide excision repair is organized into domains that promote efficient DNA repair in chromatin
Source: Genome Res. 2016 Oct;26(10):1376–87. doi: 10.1101/gr.209106.116 (PMC5052058; doi:10.1101/gr.209106.116)
Supplement: Supplemental Material [file supp_26_10_1376__index.html]

Global genome nucleotide excision repair is organized into domains that promote efficient DNA repair in chromatin — Supplemental Material 

# Global genome nucleotide excision repair is organized into domains that promote efficient DNA repair in chromatin

## Supplemental Material

- Supplemental\_Figures.docx
- Supplemental\_Methods.docx
