# Supplementary material for: Global genome nucleotide excision repair is organized into domains that promote efficient DNA repair in chromatin
Source: Genome Res. 2016 Oct;26(10):1376–87. doi: 10.1101/gr.209106.116 (PMC5052058; doi:10.1101/gr.209106.116)
Supplement: Supplemental Material [file supp_gr.209106.116_Supplemental_Figures.docx]

# **Supplemental Figures**


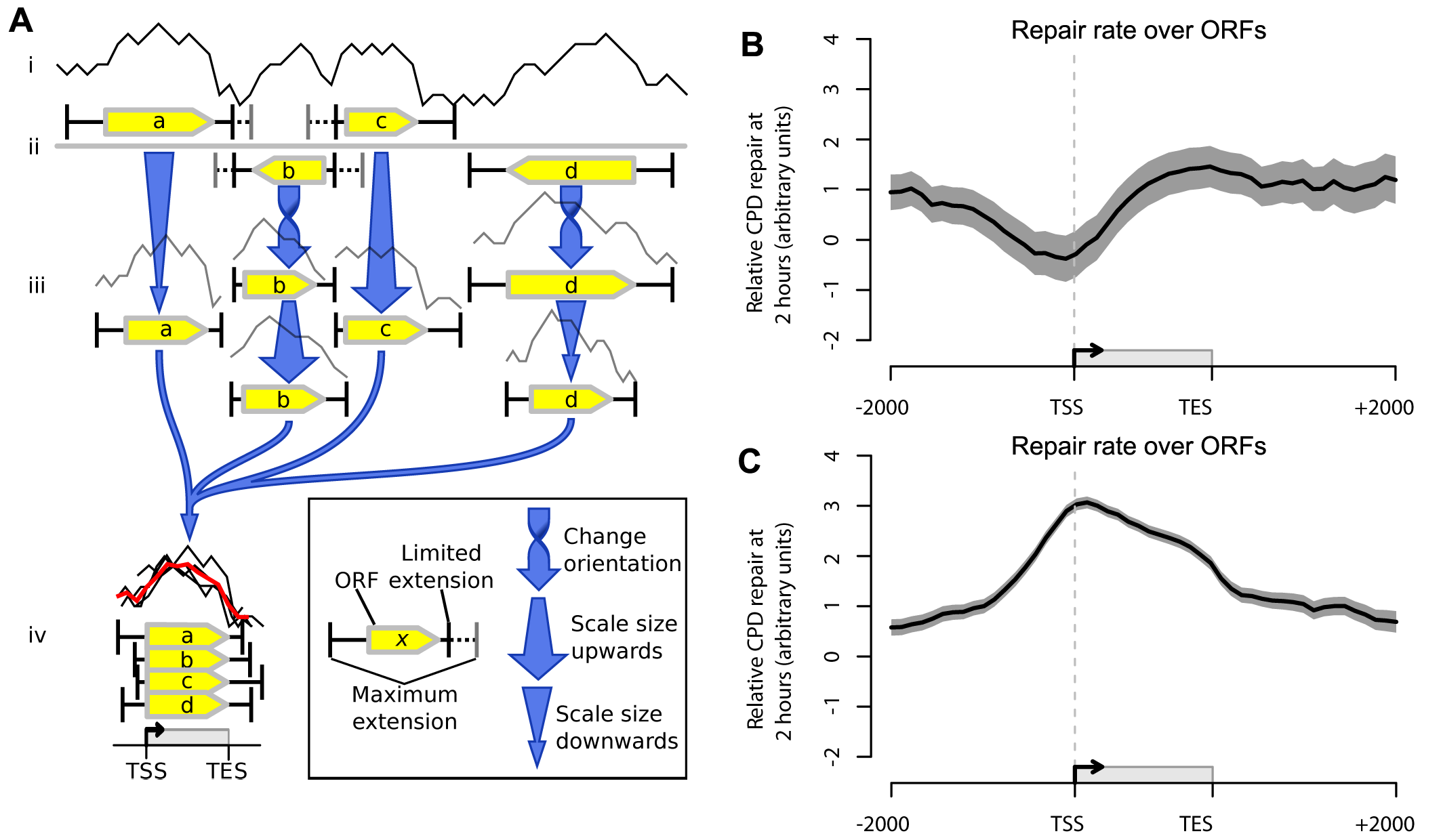


Supplemental Figure S1 – **Representation of the Sandcastle process used to overlay data from multiple ORFs to create composite plots. Enhanced rates of repair in ORFs are due to TC-NER of actively transcribed genes.** (**A**) A section of (i) data and (ii; labelled a, b, c, and d) ORFs are shown. Bars extending from the ORFs show the maximum length of extensions to incorporate surrounding data. Where these extensions overlap with adjacent ORF extensions (dotted line sections) they are both limited to a point halfway between the two, such that any given data point is plotted no more than once. (iii) Sections of data are then all oriented in the same direction and scaled up- or downwards so that each ORF covers the same length. (iv) These are then overlaid and an average of all the data sections calculated (red line). For all plots shown here ORFs from 500 to 1,500 bp in size were analysed (n = 2,974). (**B**) Relative rates of CPD removal in the lowest 15% of transcribed ORFs in wild type cells (n = 3, black). Solid lines show the mean of relative CPD repair rates, with the shaded areas highlighting the SEM. (**C**) As (B) now showing repair rates in the remaining 85% of actively transcribing ORFs. CPD levels are plotted as arbitrary units on the y-axis.


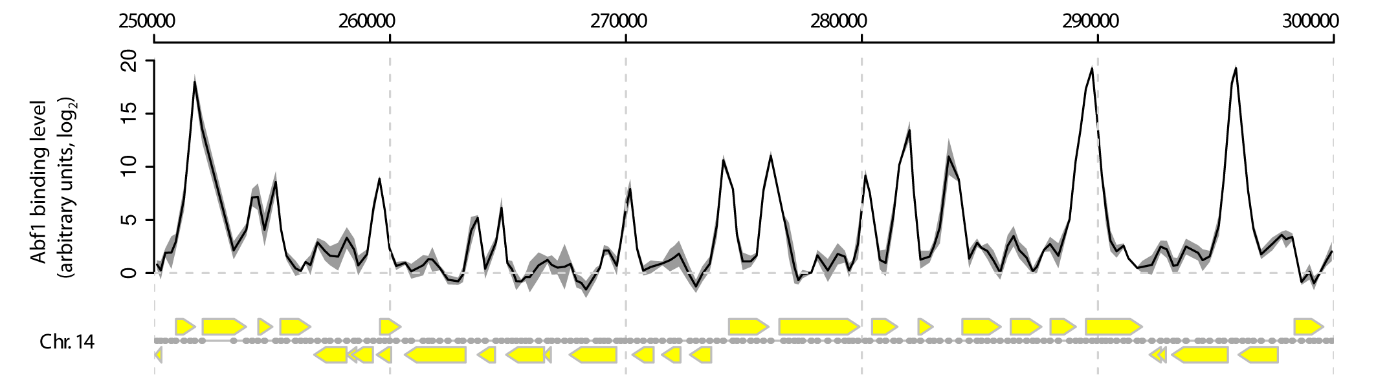


Supplemental Figure S2 - **A linear genome plot of a section of chromosome 14 showing Abf1 binding in wild type cells.** The black line shows the mean (n = 3) of Abf1 binding in the absence UV irradiation. The shading indicates the SEM. Grey dots indicate positions of microarray probes. Yellow arrows indicate ORF positions and their directions of transcription.


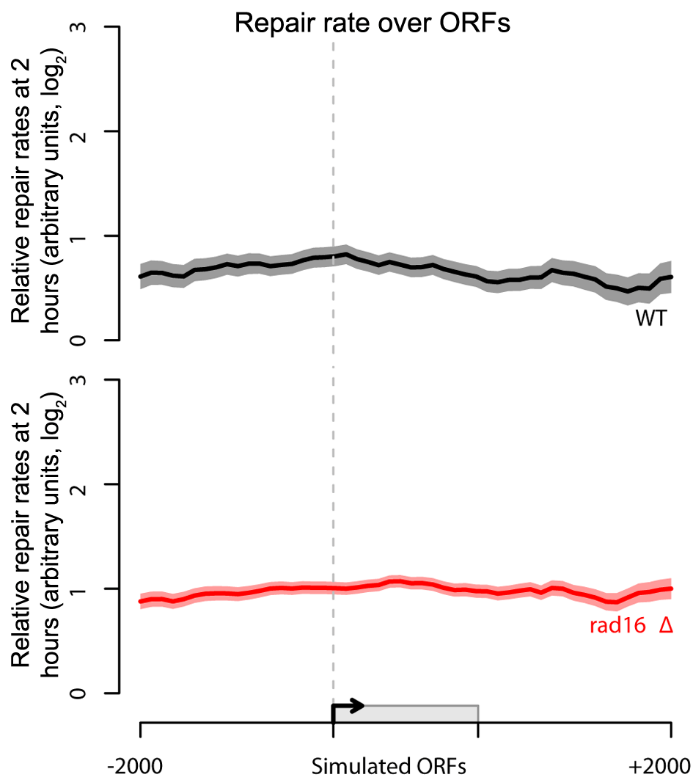


Supplemental Figure S3 – **CPD removal at randomly selected simulated ORFs shows no difference in the distribution of repair rates between the wild type and GG-NER mutant cells.** Relative rates of CPD removal around 2,974 randomly generated simulated composite ORF plots in wild type (black) and *rad16Δ* cells (red). Shaded areas show the SEM and SD, respecitvely and CPD levels are plotted as arbitrary units on the y-axis.


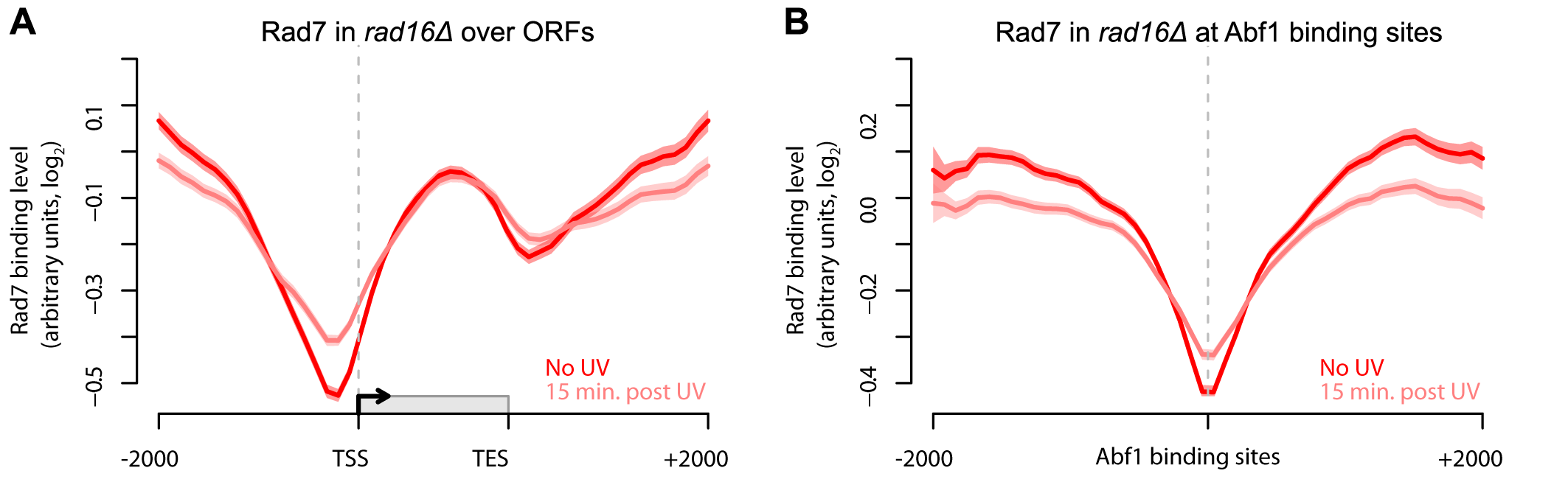


Supplemental Figure S4 – **The GG-NER proteins bind to chromatin as a complex.** (**A**) Rad7 binding data in *rad16Δ* is shown around ORF structure for unirradiated (red) and 15 min post-UV (pink) datasets. Solid lines show the mean of three datasets per time point and shaded areas show the SEM. (**B**) As (A) plotted around Abf1 binding sites.


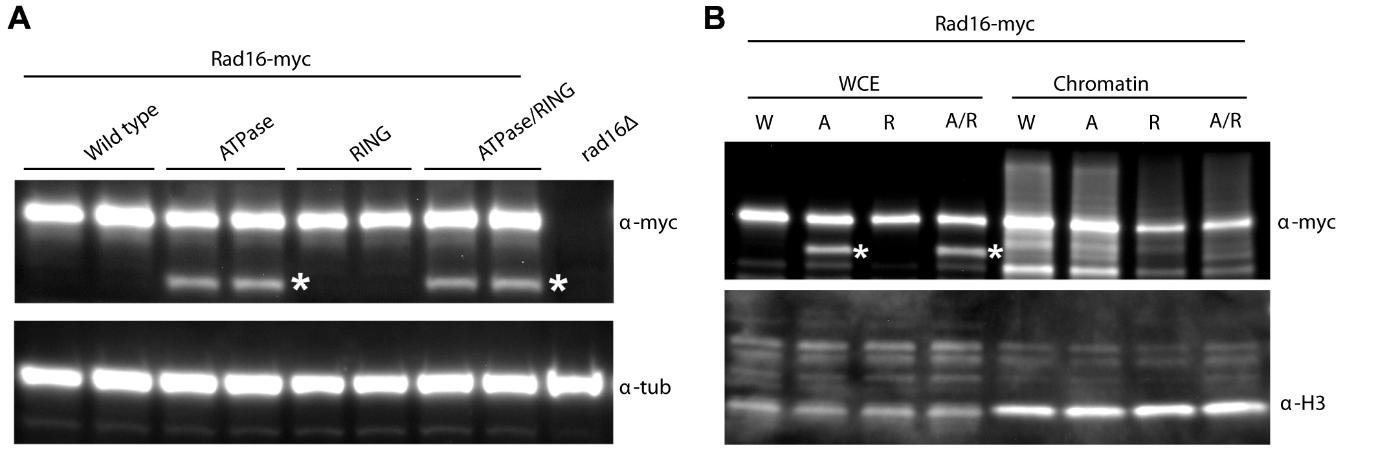


Supplemental Figure S5 – **Introducing mutations in the RING and ATPase domains does not affect full length protein production of Rad16.** (**A**) Western blot of the expression of the mutated, Myc-tagged Rad16 proteins reveals production of full length protein at normal levels (top panel). The last lane was used to include protein extracts of *rad16Δ* cells as a negative control. For each mutant a pair of two individual clones are shown here. As a loading control the blot was stripped and probed for tubulin (lower panel). The asterisks indicate the presence of minor Rad16 degradation associated with the ATPase mutation. (**B**) Western blot as A, now showing the correct association of the mutated Rad16 proteins with chromatin (top panel). W = wild type, A = ATPase mutant, R = RING mutant, A/R = ATPase/RING double mutant, WCE = Whole Cell Extract. The lower panel displays the histone H3 probed Western blot as a positive control for chromatin associated protein.


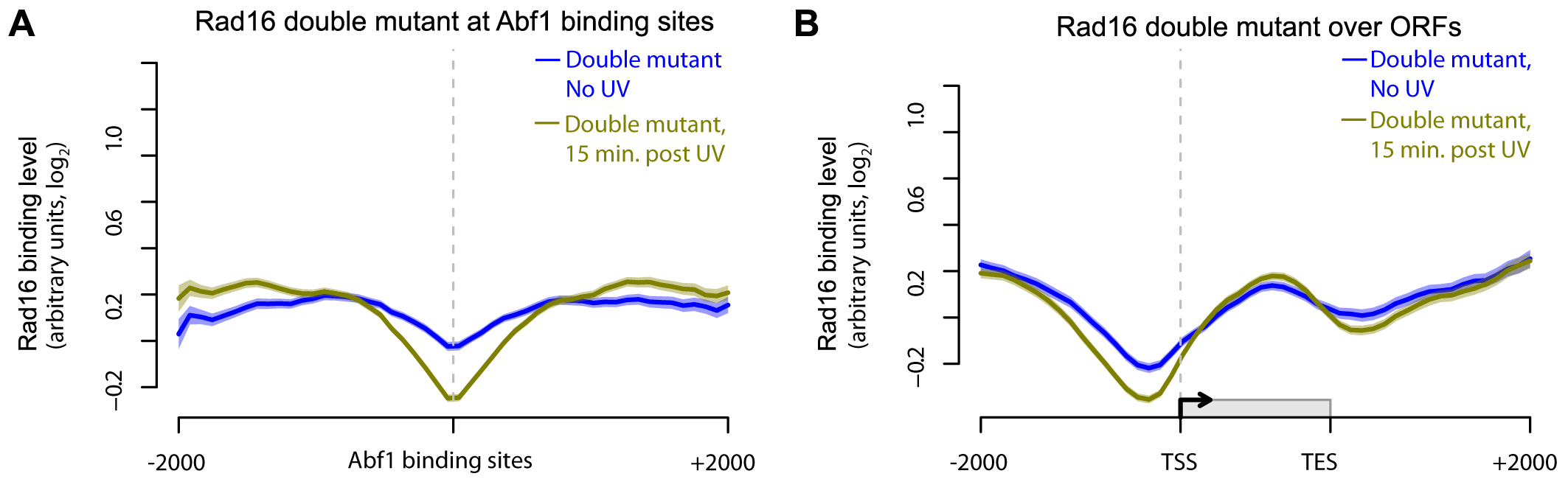


Supplemental Figure S6 – **The activity of both the ATPase and RING domain of Rad16 determine its chromatin occupancy before and after UV irradiation.** *Composite plots of Rad16 chromatin occupancy in the Rad16RING/ATPase double mutant.* Mutated Rad16RING/ATPase binding data around (A) Abf1 binding sites and (B) ORF structures in the absence of UV irradiation (dark blue) and 15 minutes after UV irradiation (green) is shown here.


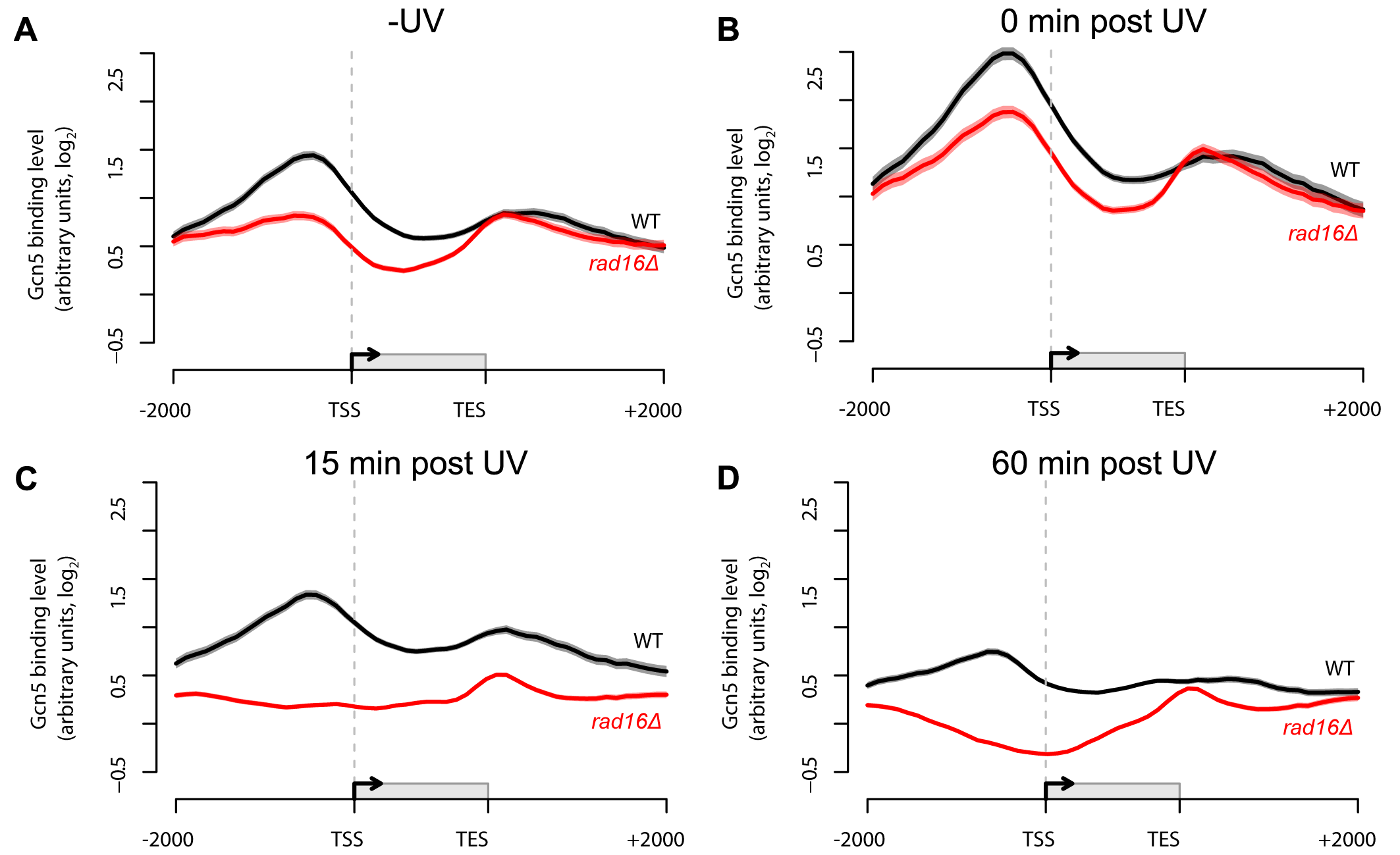


Supplemental Figure S7 – **Gcn5 is recruited to and retained in the vicinity of ABF1 binding sites in response to UV in a GG-NER-dependent manner.** Gcn5 ChIP-Chip data from Figure 6C and D is shown here split into the four different time points to highlight the GG-NER-dependent Gcn5 retention on chromatin in the following time points the (**A**) in absence of UV, (**B**) at 0 min, (**C**) 15 min and (**D**) 60 min after UV irradiation.


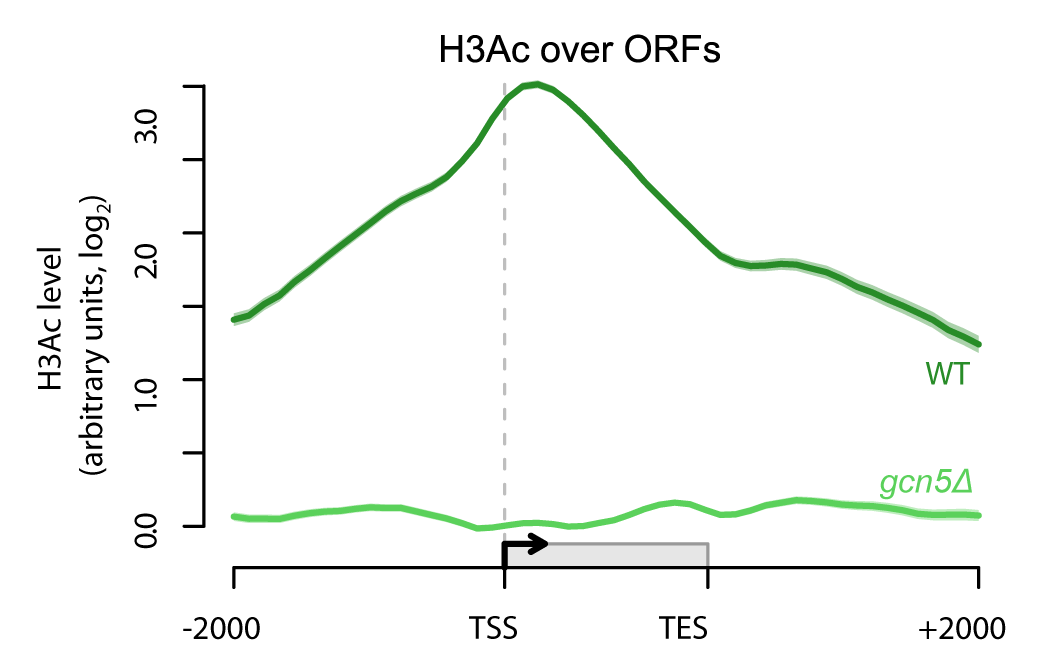


Supplemental Figure S8 – **Histone H3 acetylation induced by UV irradiation in wild type cells depends on the HAT Gcn5.** Post-UV H3Ac levels in wild type (dark green) and *gcn5Δ* (light green) around ORFs are shown here. Solid lines show the mean of three datasets. Shaded areas show the SEM.
