# Supplementary material for: Global genome nucleotide excision repair is organized into domains that promote efficient DNA repair in chromatin
Source: Genome Res. 2016 Oct;26(10):1376–87. doi: 10.1101/gr.209106.116 (PMC5052058; doi:10.1101/gr.209106.116)
Supplement: Supplemental Material [file supp_gr.209106.116_Supplemental_Methods.docx]

# **Supplemental methods**

## UV irradiation, yeast cell culture and crosslinking

Yeast cells were grown and UV irradiated as described previously (Yu et al. 2011). After the indicated repair time in YPD, 100 mL of cells were treated with formaldehyde to a final concentration of 1% for between 10-40 mins at room temperature to crosslink protein-DNA complexes. Crosslinking was stopped with the addition of 5.5 mL Glycine (2.5 M) to 100 mL cells. Cells were collected by centrifugation and resuspended in cold PBS. For Rad7 affinity capture using ChIP, a double crosslinking method is performed using 2x10^9^ cells collected by centrifugation and washed once with cold PBS. Crosslinking was carried out in two steps; first protein-protein crosslinking was performed using DMA (Dimethyl adipmidatedihydrochloride). Cells were resuspended in 10 mL of 10 mM and 0.25 % DMSO in cold PBS and incubated at room temperature for 45 min with mixing. Secondly, the cells were collected by centrifugation and washed 3 times with PBS and resuspended in 100 mL YPD. Finally, DNA-protein crosslinking was performed by addition of formaldehyde to a final concentration of 1%. The cell suspension was incubated for 45 min at room temperature.

## Chromatin preparation

Chromatin extracts were prepared as described previously (Teng et al. 2011; Yu et al. 2011). Briefly, cells were washed two times in cold PBS, followed by a wash step with cold FA/SDS (50 mM HEPES KOH pH 7.5, 150 mM NaCl, 1 mM EDTA, 1% Triton X-100, 0.1% NaDeoxycholate, 0.1% SDS, 1 mM PMSF). The cells were collected by centrifugation and prepared for lysis by bead beating in FA/SDS (+PMSF) using 0.5 mL of glass beads. The whole cell extract was then sonicated with a Bioruptor (Diagenode) as described previously (Yu et al. 2011), after which the chromatin extra was collected by centrifugation.

## Chromatin Immunoprecipitation

ChIP was performed as described previously (Yu et al. 2011; Powell et al. 2015). In short, pre-washed pan mouse or anti-rabbit IgG Dynabeads were incubated with the respective antibody for 30 min at 30°C (2.5 µg of mouse anti-Myc (9E11, Abcam) antibody, 2.5 µL of rabbit anti-acetyl histone H3 (at K9 and K14, Upstate Biotechnology) or 20 µL anti-Abf1 antibody (yC-20, #sc-6679, Santa Cruz Biotechnology). Dynabeads were collected, washed and resuspended in 50 µL of PBS-BSA (0.1%) per sample. 30 µL of 10x PBS-BSA (10 mg/mL) and 100 µL of sonicated DNA were added to each sample containing the Dynabeads, and the final volume made up to 300 µL with PBS. Samples were incubated at 21°C for 3 hours at 1300 rpm in an Eppendorf thermomixer. Following incubation, samples underwent a series of washes after which DNA was eluted from the Dynabeads in 125 µL of pronase buffer (25 mM Tris pH 7.5, 5 mM EDTA, 0.5% SDS) at 65°C at 900 rpm for 30 min. Pronase was added to each sample and incubated at 65°C in a water bath overnight. 50 µL of chromatin were taken as input samples and were treated with pronase and incubated overnight as the IP samples. The IP and input samples were treated with 5ul of DNase-free RNase A (10 mg/mL) for 1 hour at 37°C prior to DNA purification using the PureLink Quick PCR Purification Kit (Invitrogen) and eluted with 50 mL elution buffer.

## DNA preparation and IP for CPD detection

DNA was prepared and sonicated as described previously (Teng et al. 2011). IP was conducted as described in previous section (Chromatin Immunopreciptation) with the exception of using an antibody for CPD IP (2 µg per sample of Anti-Thymine Dimer clone KTM53, Kamiya Biomedical Company). Following IP, all samples were processed the same way to microarray.

## Removal of CPDs prior to microarray preparation and real-time PCR

CPDs need to be removed from the UV treated samples prior to PCR amplification and microarray hybridisation. The PreCR DNA repair kit (New England Biolabs) removes many DNA damages including CPDs. 40 µL of IP and IN sample were processed for repair using the PreCR repair kit as per the manufacturer’s instructions. Following repair, DNA was retrieved using phenol-chloroform extraction and ethanol precipitation and resuspended in 10 µL.

## DNA preparation and micro array hybridization

Samples were prepared for microarray hybridisation as detailed in the Agilent Technologies Yeast ChIP on chip protocol (Agilent Technologies Yeast ChIP-on-chip Analysis Protocol, version 9.2) and described previously (Powell et al, 2015). Briefly, DNA fragments are blunt-ended and a common linker sequence is ligated to their ends for two steps of ligation-mediated PCR amplification. The DNA concentration was measured with the NanoDrop spectrophotometer and adjusted to 150 ng/µL with H_2_O. 10.5 µL of IP and IN samples were differentially labelled with Alexa Fluor 5 and 3 dyes respectively, using the BioPrime Total Genomic Labelling System (Invitrogen). Labelling efficiency was determined using the MicroArray Measurement Module on the NanoDrop ND-1000 Spectrophotometer. The IP and input samples were combined and 110 µL hybridisation mixture applied to each Agilent yeast microarray. Mixtures were hybridised for 24 hours at 65°C. After hybridisation the microarrays were washed twice and scanned as described in the Agilent Technologies Yeast ChIP on chip protocol. The scanned image was processed with Agilent Feature Extraction software, which converts fluorescence intensities into numerical values for analysis. Analysis of the data was conducted using Sandcastle (Bennett et al. 2015) in R version 3.2.4 , which uses log_2_ values of IP:input ratios.

## Data Normalisation

Data from each experiment were normalised using the 'normalise' function in Sandcastle. The full Sandcastle normalisation procedure was applied to the individual protein binding and H3Ac datasets. This performs quantile normalisation on each set of replicates (for each time point), and then shifts and scales all datasets (combining all time points) together. This allows comparisons to be made between the binding levels of the protein/H3Ac at the different time points. Note that this procedure transforms the data so that for each set of protein/H3Ac datasets the values are comparable between the different time points analysed, but are not comparable between the different proteins/H3Ac. Only the quantile normalisation step was applied to each set of replicates of the CPD datasets, because these data are not suitable for the full Sandcastle normalisation procedure. For this reason the relative DNA repair rates calculated are plotted using arbitrary units.

## Data Analysis

The composite plots shown in this paper were created using the 'profilePlot' function of Sandcastle. Plots around Abf1 binding sites were created using peaks detected in the no UV treatment Abf1 binding datasets using the 'enrichmentDetection' function. Plots over ORFs were created using data downloaded from the Ensembl databases using the 'loadAnnotation' function. Full details of these procedures are described in the Sandcastle publication and associated documentation (Bennett et al. 2015).

## References

Bennett M, Evans KE, Yu S, Teng Y, Webster RM, Powell J, Waters R, Reed SH. 2015. Sandcastle: software for revealing latent information in multiple experimental ChIP-chip datasets via a novel normalisation procedure. *Sci Rep* **5**: 13395.

Powell JR, Bennett MR, Evans KE, Yu S, Webster RM, Waters R, Skinner N, Reed SH. 2015. 3D-DIP-Chip: a microarray-based method to measure genomic DNA damage. *Sci Rep* **5**.

Teng Y, Bennett M, Evans KE, Zhuang-Jackson H, Higgs A, Reed SH, Waters R. 2011. A novel method for the genome-wide high resolution analysis of DNA damage. *Nucleic acids research* **39**(2): e10.

Yu S, Teng Y, Waters R, Reed SH. 2011. How chromatin is remodelled during DNA repair of UV-induced DNA damage in Saccharomyces cerevisiae. *PLoS genetics* **7**(6): e1002124.
